# Supplementary material for: Integrated genomic and functional analyses of human skin-associated Staphylococcus reveals extensive inter- and intra-species diversity
Source: bioRxiv. 2023 Jun 23:2023.06.22.546190. Preprint. [Version 1] doi: 10.1101/2023.06.22.546190 (PMC10370188; doi:10.1101/2023.06.22.546190)
Supplement: Supplement 1 [file media-1.pdf]

Supporting Information for

Integrated genomic and functional analyses of human skin-associated *Staphylococcus* reveals extensive inter- and intra-species diversity

Payal Joglekar [a], Sean Conlan [a], Shih-Queen Lee-Lin [a], Clay Deming [a], Sara Saheb Kashaf [a], NISC Comparative Sequencing Program [b], Heidi H. Kong [c], Julia A. Segre [a†]

[a] Microbial Genomics Section, Translational and Functional Genomics Branch, NHGRI, NIH, Bethesda, Maryland, USA

[b] NIH Intramural Sequencing Center, NHGRI, NIH, Rockville, Maryland, USA

[c] Cutaneous Microbiome and Inflammation Section, NIAMS, NIH, Bethesda, Maryland, USA

[†] Corresponding author

Julia A. Segre

Building 49, Room 4a26,

49 Convent Dr. 4442,

Bethesda, MD 20892-4442

(301) 402-2314

jsegre@nhgri.nih.gov

This PDF file includes:

- Figures S1 to S10
- Tables S1, S5, S8 and S9
- Legends for Datasets S2, S3, S4, S6, S7, S10
- SI References

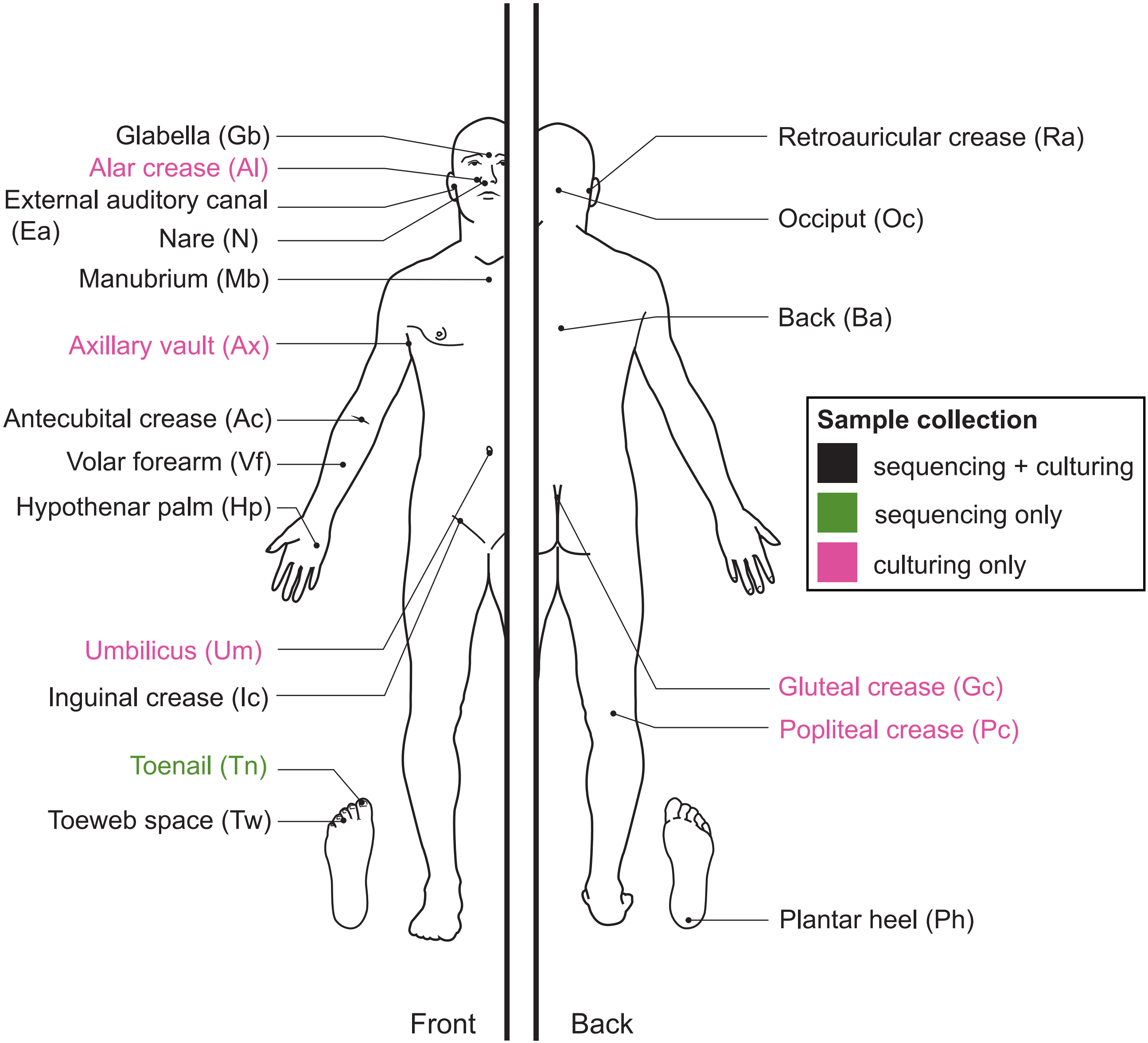

Glabella (Gb)
Alar crease (Al)
External auditory canal (Ea)
Nare (N)
Manubrium (Mb)
Axillary vault (Ax)
Antecubital crease (Ac)
Volar forearm (Vf)
Hypothenar palm (Hp)
Umbilicus (Um)
Inguinal crease (Ic)
Toenail (Tn)
Toeweb space (Tw)
Retroauricular crease (Ra)
Occiput (Oc)
Back (Ba)
Sample collection
sequencing + culturing
sequencing only
culturing only
Gluteal crease (Gc)
Popliteal crease (Pc)
Plantar heel (Ph)
Front
Back

Figure S1. **Body sites used for collecting samples from healthy volunteers for 16S rRNA amplicon sequencing and culturing of staphylococcal isolates**.

Sites shown in black represent those for which both types of samples were collected. Toenail samples (green) were only collected for sequencing. Umbilicus, Gluteal crease, and Popliteal crease samples (all pink) were collected for culturing alone. Abbreviation shown in a bracket next to each body site were used to denote the site throughout the manuscript.

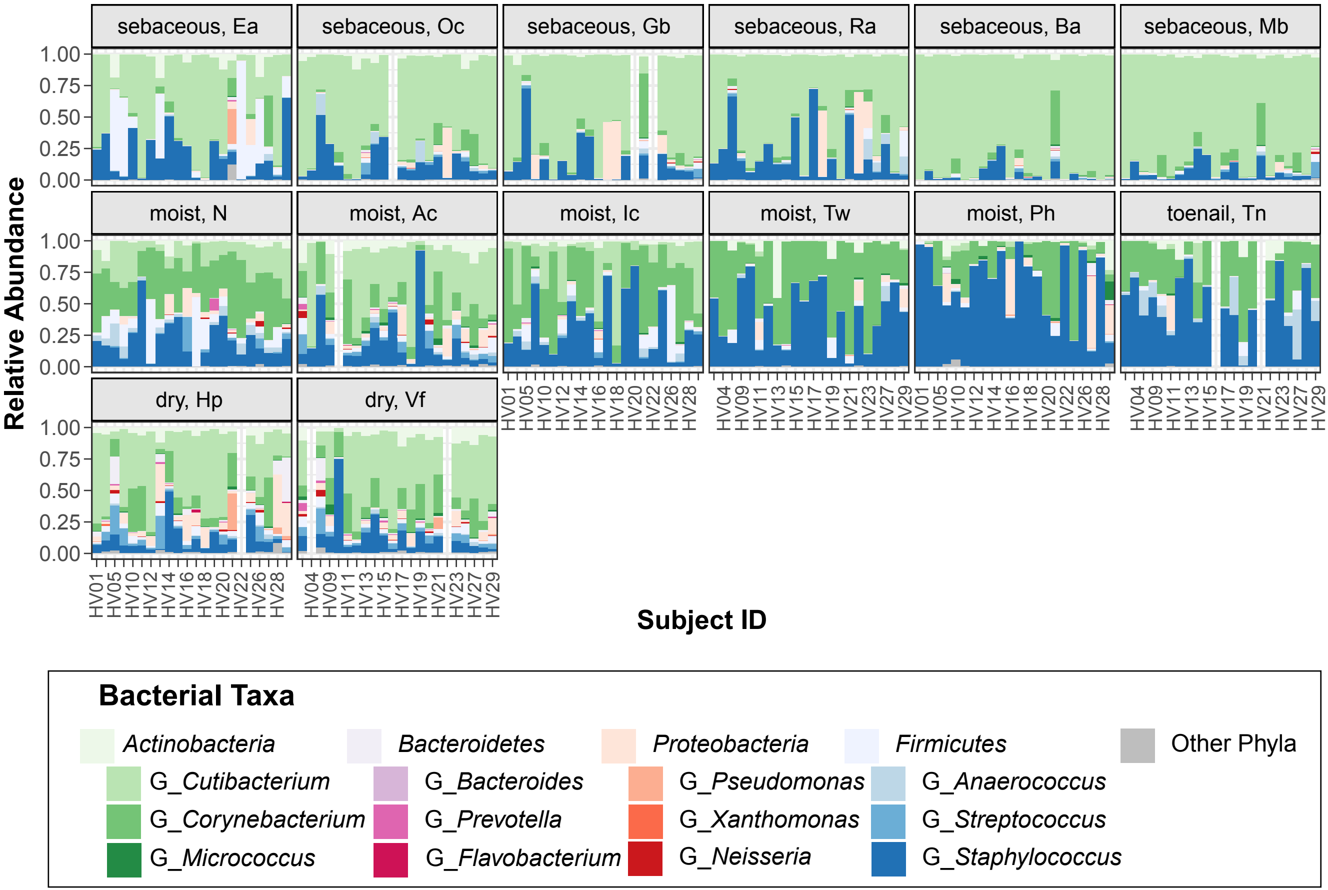

sebaceous, Ea
sebaceous, Oc
sebaceous, Gb
sebaceous, Ra
sebaceous, Ba
sebaceous, Mb
moist, N
moist, Ac
moist, Ic
moist, Tw
moist, Ph
toenail, Tn
dry, Hp
dry, Vf
Relative Abundance
0.00
0.25
0.50
0.75
1.00
HV01 HV05 HV10 HV12 HV14 HV16 HV18 HV20 HV22 HV26 HV28
HV04 HV09 HV11 HV13 HV15 HV17 HV19 HV21 HV23 HV27 HV29
Subject ID
Bacterial Taxa
Actinobacteria
G_Cutibacterium
G_Corynebacterium
G_Micrococcus
Bacteroidetes
G_Bacteroides
G_Prevotella
G_Flavobacterium
Proteobacteria
G_Pseudomonas
G_Xanthomonas
G_Neisseria
Firmicutes
G_Anaerococcus
G_Streptococcus
G_Staphylococcus
Other Phyla

Figure S2. **Bacterial diversity on healthy human skin represented by major phyla and genera colonizing distinct body sites.**

Barplots display the relative abundance of major bacterial taxa at various body sites as displayed by facets. Colors represent taxa as shown in the accompanying legend. Each bar represents one volunteer. Empty bars represent missing data. Refer to Fig. S1 for body site abbreviation.

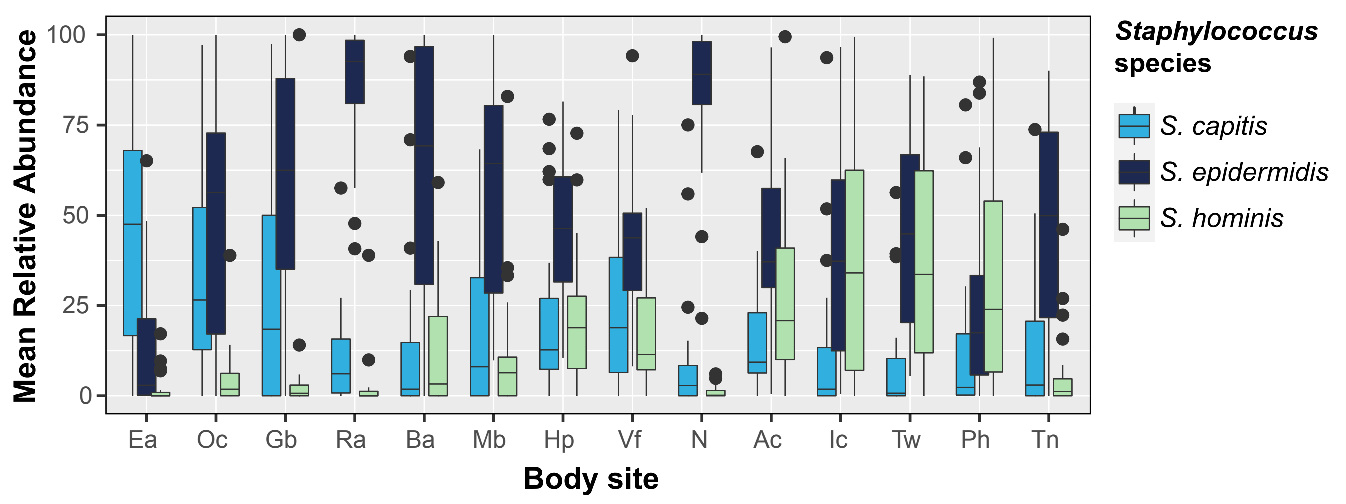
Mean Relative Abundance
100
75
50
25
0
Ea
Oc
Gb
Ra
Ba
Mb
Hp
Vf
N
Ac
Ic
Tw
Ph
Tn
Body site
Staphylococcus species
S. capitis
S. epidermidis
S. hominis

Figure S3. Mean relative abundance (MRA) of the three most prominent species within staphylococcal communities

Boxplots represents the MRA of the three staphylococcal species across all body sites as shown on the x-axis. Boxplot colors represent individual species. The center black line within each boxplot represents the median value, with edges showing the first and third quartiles. Refer to Fig. S1 for body site abbreviation.

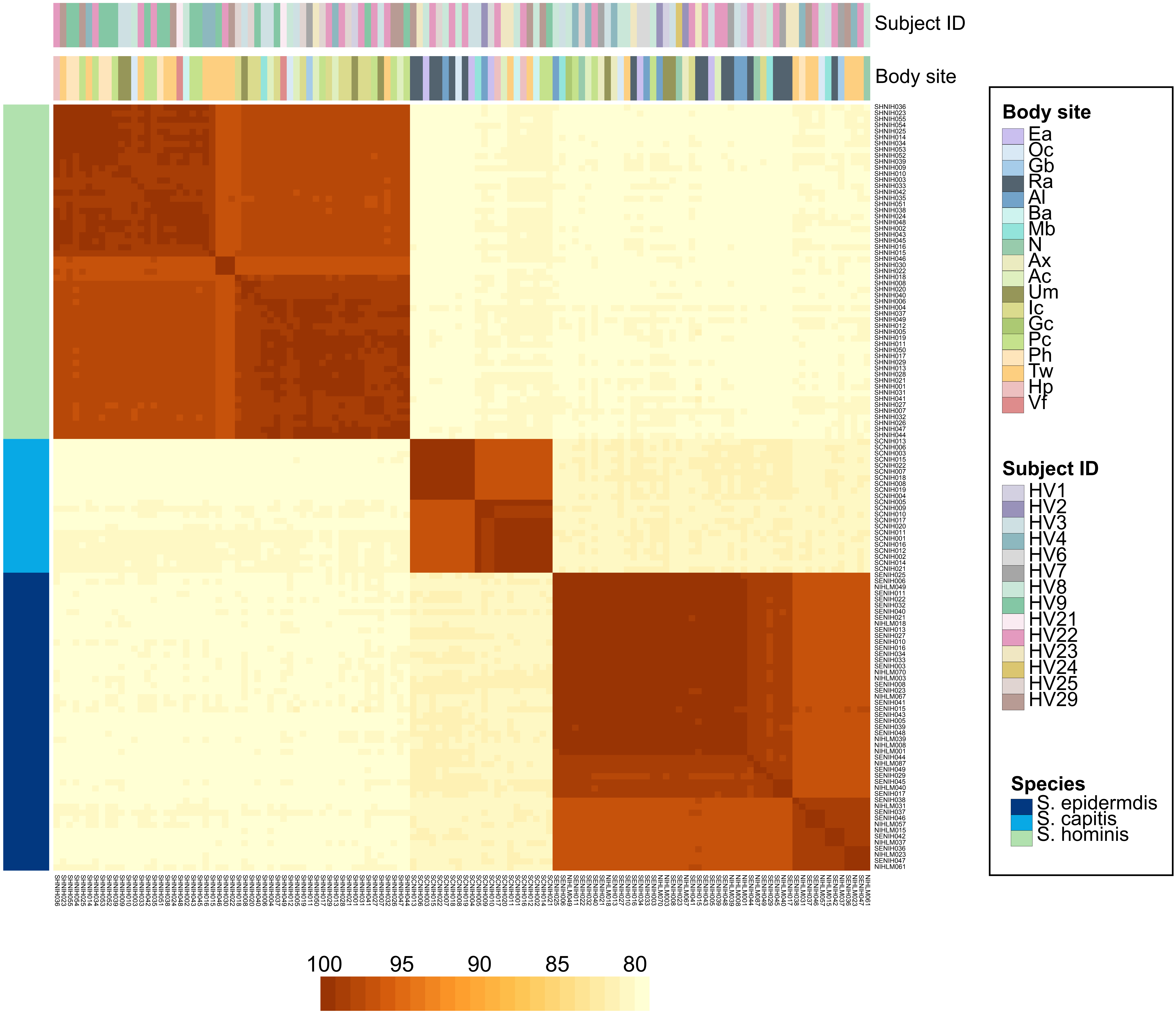
Subject ID
Body site
Body site
Ea
Oc
Gb
Ra
Al
Ba
Mb
N
Ax
Ac
Um
Ic
Gc
Pc
Ph
Tw
Hp
Vf
Subject ID
HV1
HV2
HV3
HV4
HV6
HV7
HV8
HV9
HV21
HV22
HV23
HV24
HV25
HV29
Species
S. epidermdis
S. capitis
S. hominis
100
95
90
85
80

Figure S4. **Heat-map of pairwise fastANI comparison of de-replicated, non-clonal genomes of *S. epidermidis, S. capitis*, and *S. hominis*.**

Colored bars on the top denote the healthy volunteer and body site of isolation of each labeled genome. Species are denoted by the side bar on the left. Refer to Fig. S1 for body site details.

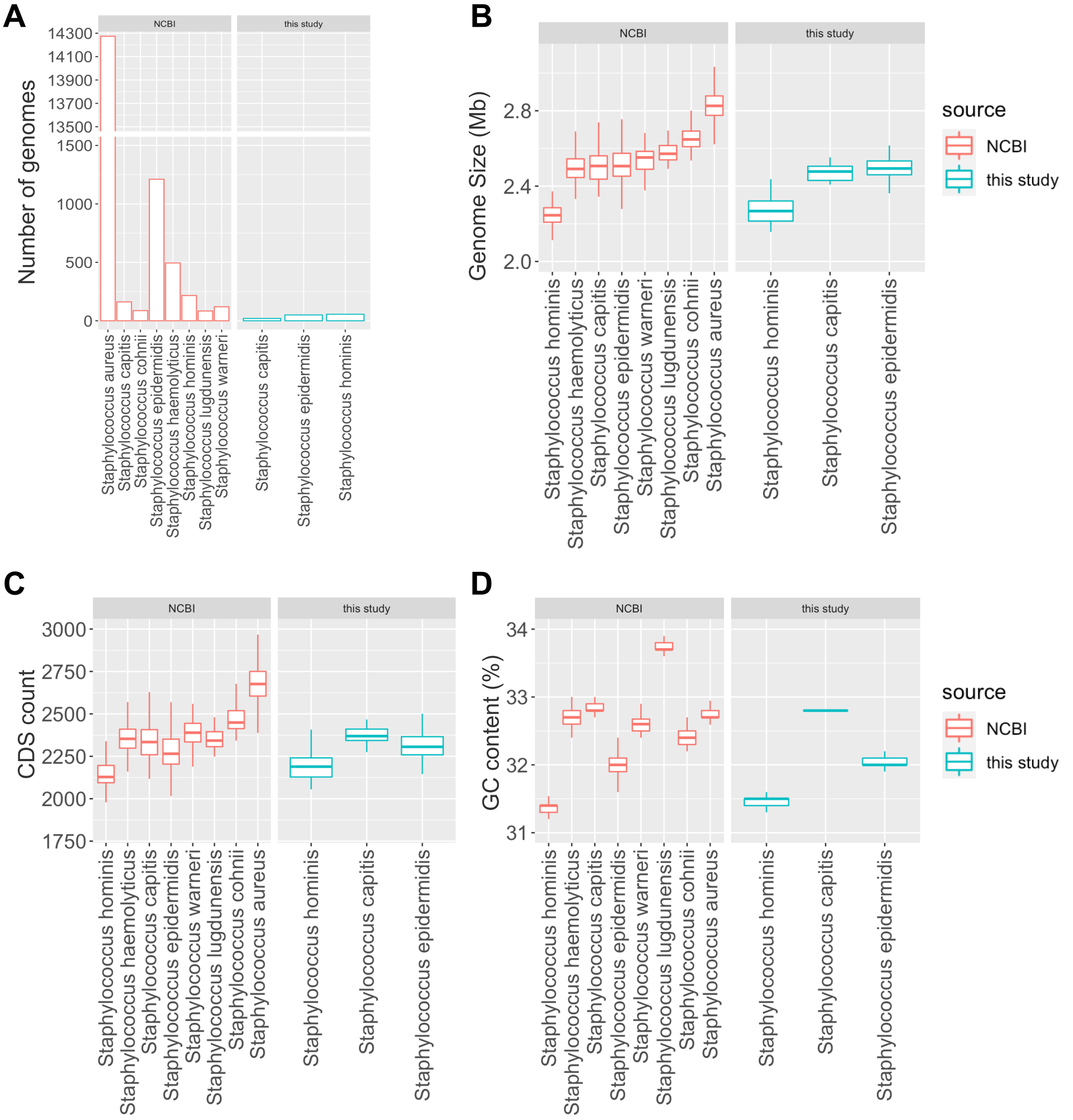

A
NCBI
this study
Number of genomes
14300
14100
13900
13700
13500
1500
1000
500
0
Staphylococcus aureus
Staphylococcus capitis
Staphylococcus cohnii
Staphylococcus epidermidis
Staphylococcus haemolyticus
Staphylococcus hominis
Staphylococcus lugdunensis
Staphylococcus warneri
Staphylococcus capitis
Staphylococcus epidermidis
Staphylococcus hominis
B
NCBI
this study
Genome Size (Mb)
2.8
2.4
2.0
Staphylococcus hominis
Staphylococcus haemolyticus
Staphylococcus capitis
Staphylococcus epidermidis
Staphylococcus warneri
Staphylococcus lugdunensis
Staphylococcus cohnii
Staphylococcus aureus
Staphylococcus hominis
Staphylococcus capitis
Staphylococcus epidermidis
source
NCBI
this study
C
NCBI
this study
CDS count
3000
2750
2500
2250
2000
1750
Staphylococcus hominis
Staphylococcus haemolyticus
Staphylococcus capitis
Staphylococcus epidermidis
Staphylococcus warneri
Staphylococcus lugdunensis
Staphylococcus cohnii
Staphylococcus aureus
Staphylococcus hominis
Staphylococcus capitis
Staphylococcus epidermidis
D
NCBI
this study
GC content (%)
34
33
32
31
Staphylococcus hominis
Staphylococcus haemolyticus
Staphylococcus capitis
Staphylococcus epidermidis
Staphylococcus warneri
Staphylococcus lugdunensis
Staphylococcus cohnii
Staphylococcus aureus
Staphylococcus hominis
Staphylococcus capitis
Staphylococcus epidermidis
source
NCBI
this study

Figure S5. **Boxplot representing genome characteristics of isolates used in the current study and comparing them to species-specific genomes present in NCBI.**
(A) Number of genomes of different species that were either present in the NCBI or were sequenced for this study. **(B)** Average genome sizes of each species. **(C)** Average number of protein-coding genes (CDS) that were present in the genome of each species. **(D)** Percent GC content of each species.
The center line within each boxplot represents the median value, with edges showing the first and third quartiles.

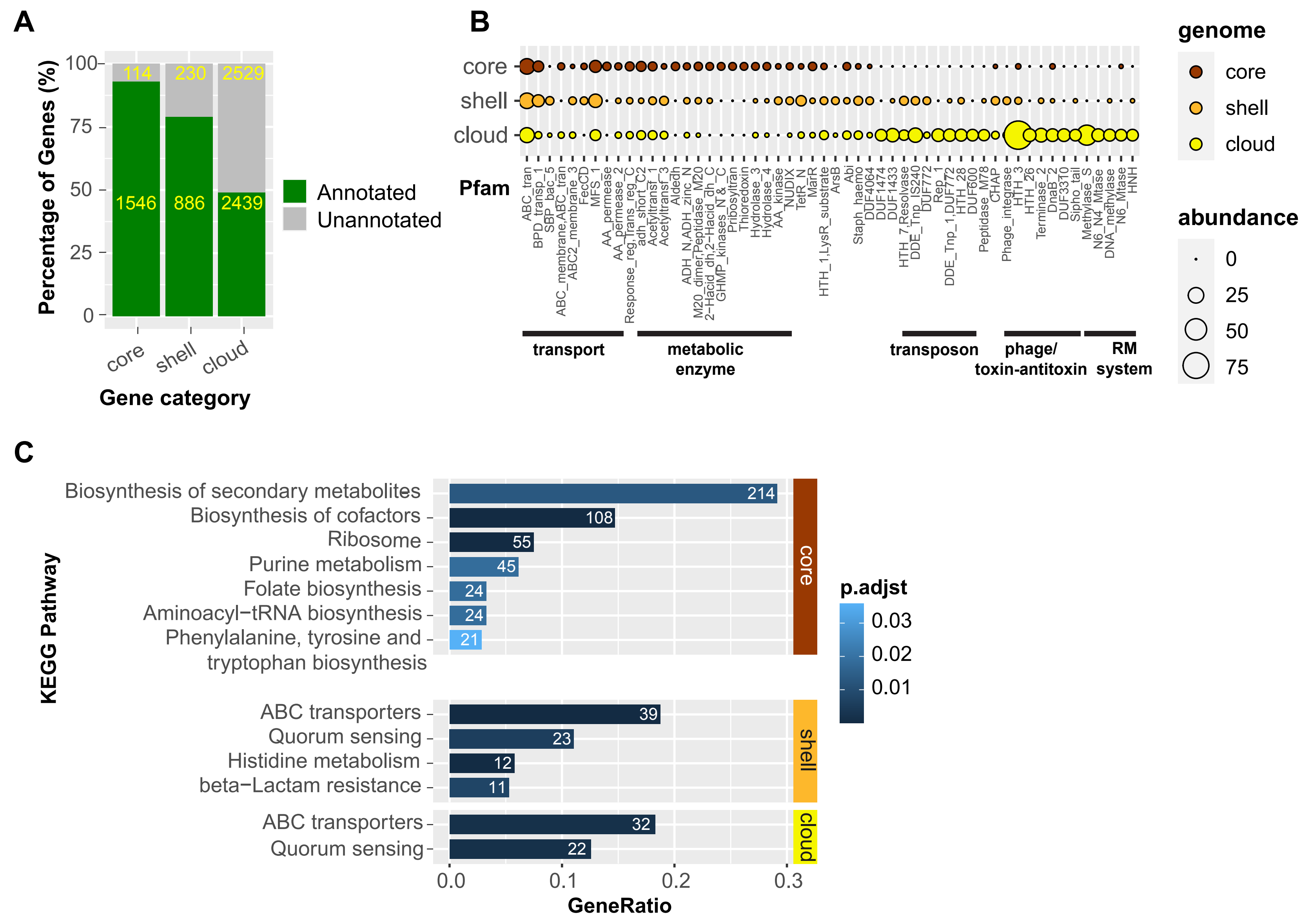
A
Percentage of Genes (%)
100
75
50
25
0
114
230
2529
1546
886
2439
core
shell
cloud
Gene category
Annotated
Unannotated
B
core
shell
cloud
Pfam
ABC_tran
BPD_transp_1
SBP_bac_5
ABC_membrane,ABC_tran
ABC2_membrane_3
FecCD
MFS_1
AA_permease
AA_permease_2
Response_reg,Trans_reg_C
adh_short_C2
Acetyltransf_1
Acetyltransf_3
Aldedh
ADH_N,ADH_zinc_N
M20_dimer,Peptidase_M20
2-Hacid_dh,2-Hacid_dh_C
GHMP_kinases_N&_C
Pribosyltran
Thioredoxin
Hydrolase_3
Hydrolase_4
AA_kinase
NUDIX
TetR_N
MarR
HTH_1,LysR_substrate
ArsB
Abi
Staph_haemo
DUF4064
DUF1474
DUF1433
HTH_7,Resolvase
DDE_Tnp_IS240
DUF772
Rep_1
DDE_Tnp_1,DUF772
HTH_28
DUF600
Peptidase_M78
CHAP
Phage_integrase
HTH_3
HTH_26
Terminase_2
DnaB_2
DUF3310
Sipho_tail
Methylase_S
N6_N4_Mtase
DNA_methylase
N6_Mtase
HNH
transport
metabolic enzyme
transposon
phage/ toxin-antitoxin
RM system
genome
core
shell
cloud
abundance
0
25
50
75
C
KEGG Pathway
Biosynthesis of secondary metabolites
Biosynthesis of cofactors
Ribosome
Purine metabolism
Folate biosynthesis
Aminoacyl−tRNA biosynthesis
Phenylalanine, tyrosine and tryptophan biosynthesis
ABC transporters
Quorum sensing
Histidine metabolism
beta−Lactam resistance
ABC transporters
Quorum sensing
214
108
55
45
24
24
21
39
23
12
11
32
22
core
shell
cloud
0.0
0.1
0.2
0.3
GeneRatio
p.adjst
0.03
0.02
0.01

Figure S6. **Functional annotation of genus pan-genome.**

(A) Percentage of annotated genes shown by genus pan-genomic category. Actual number of genes in each group are shown in yellow inside each bar. **(B)** Combined data showing top 20 most represented Pfam domains in each pan-genomic category. Size of the bubbles represents the actual number of genes carrying the Pfam domain annotation within each category.

(C) KEGG pathway enrichment analysis of genes in each category using KEGG KO identifiers.

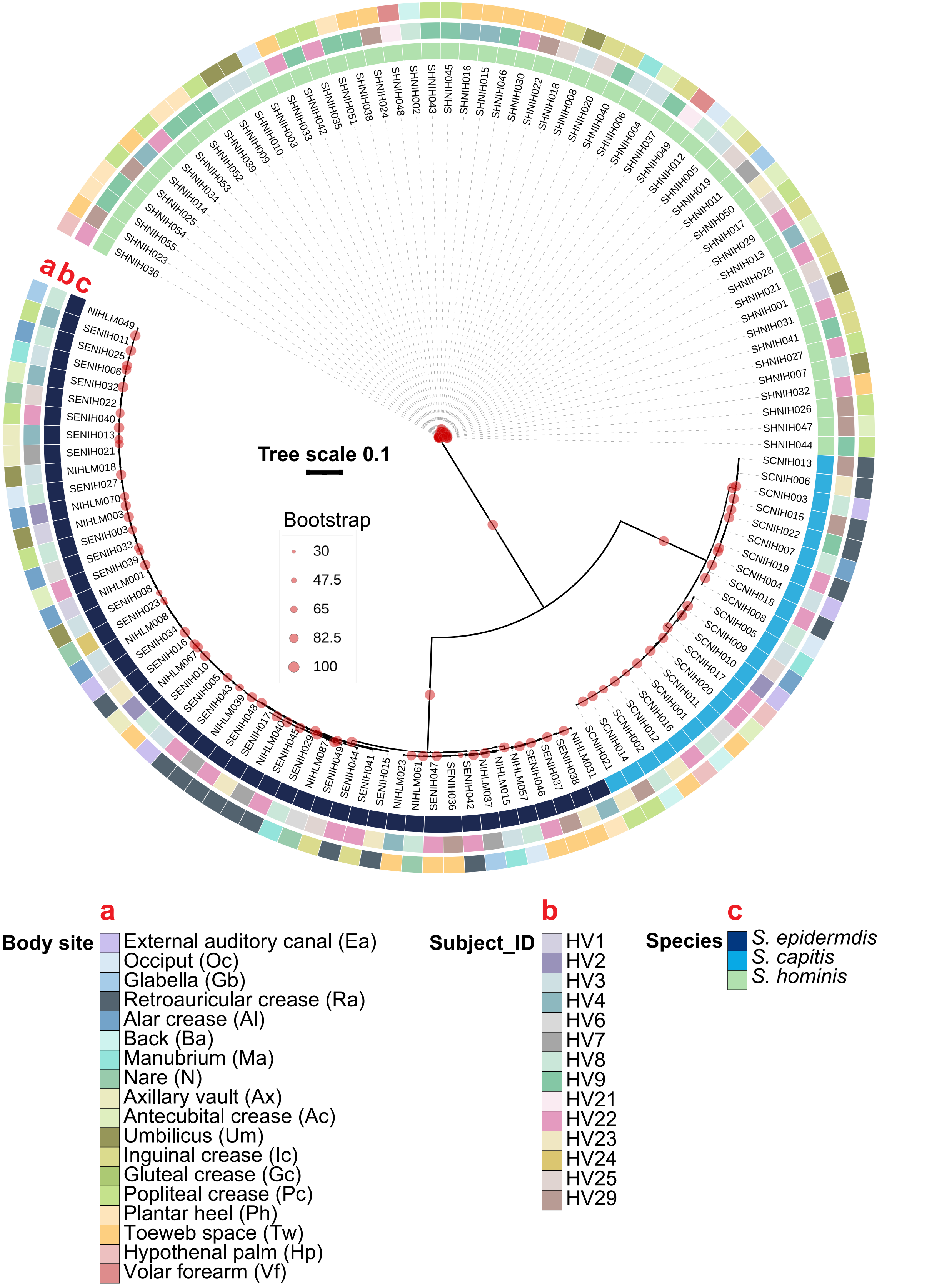

a b c
Tree scale 0.1
Bootstrap
30
47.5
65
82.5
100
a
Body site
External auditory canal (Ea)
Occiput (Oc)
Glabella (Gb)
Retroauricular crease (Ra)
Alar crease (Al)
Back (Ba)
Manubrium (Ma)
Nare (N)
Axillary vault (Ax)
Antecubital crease (Ac)
Umbilicus (Um)
Inguinal crease (Ic)
Gluteal crease (Gc)
Popliteal crease (Pc)
Plantar heel (Ph)
Toeweb space (Tw)
Hypothenal palm (Hp)
Volar forearm (Vf)
b
Subject_ID
HV1
HV2
HV3
HV4
HV6
HV7
HV8
HV9
HV21
HV22
HV23
HV24
HV25
HV29
c
Species
S. epidermdis
S. capitis
S. hominis

Figure S7. **Phylogenetic tree of all 126 staphylococcal genomes based on the polymorphic sites detected in sequence alignment of select core genes (N = 665).** Bootstrap values of at least 80 percent are shown with red dots. For each species-level phylogenetic tree shown in Figure 5, the other two species served as an outgroup for rooting the tree based on this genus-level tree. Body site, healthy volunteer, and species of each genome are shown as sidebars. Refer to Fig. S1 for body site details.

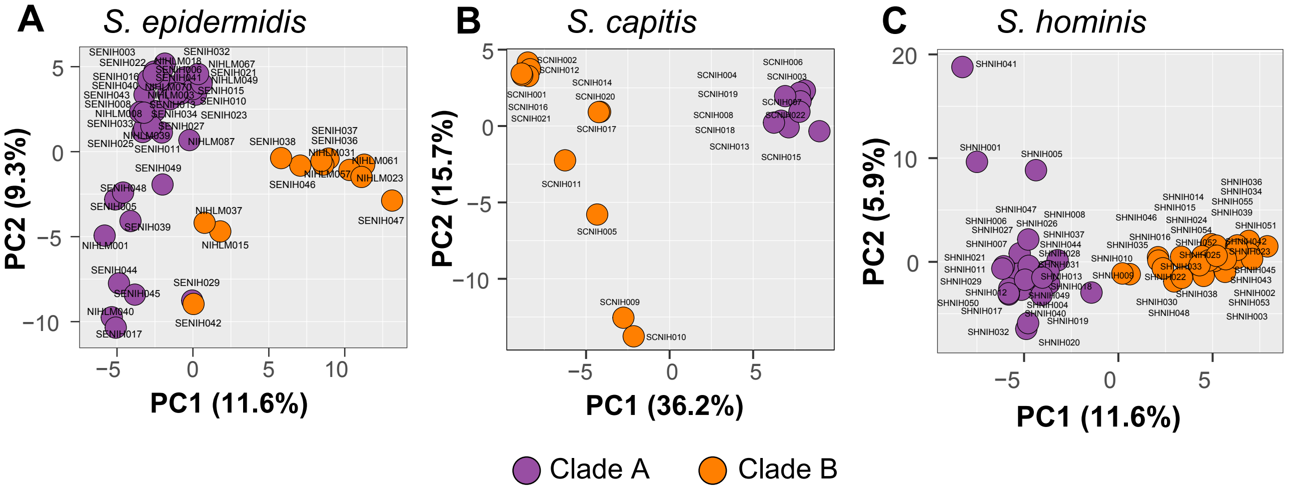

A
S. epidermidis
PC1 (11.6%)
PC2 (9.3%)
B
S. capitis
PC1 (36.2%)
PC2 (15.7%)
C
S. hominis
PC1 (11.6%)
PC2 (5.9%)
Clade A
Clade B

Figure S8. Phylogenetic tree of all 126 staphylococcal genomes based on the polymorphic sites detected in sequence alignment of select core genes (N = 665). Bootstrap values of at least 80 percent are shown with red dots. For each species-level phylogenetic tree shown in Figure 5, the other two species served as an outgroup for rooting the tree based on this genus-level tree. Body site, healthy volunteer, and species of each genome are shown as sidebars. Refer to Fig. S1 for body site details.

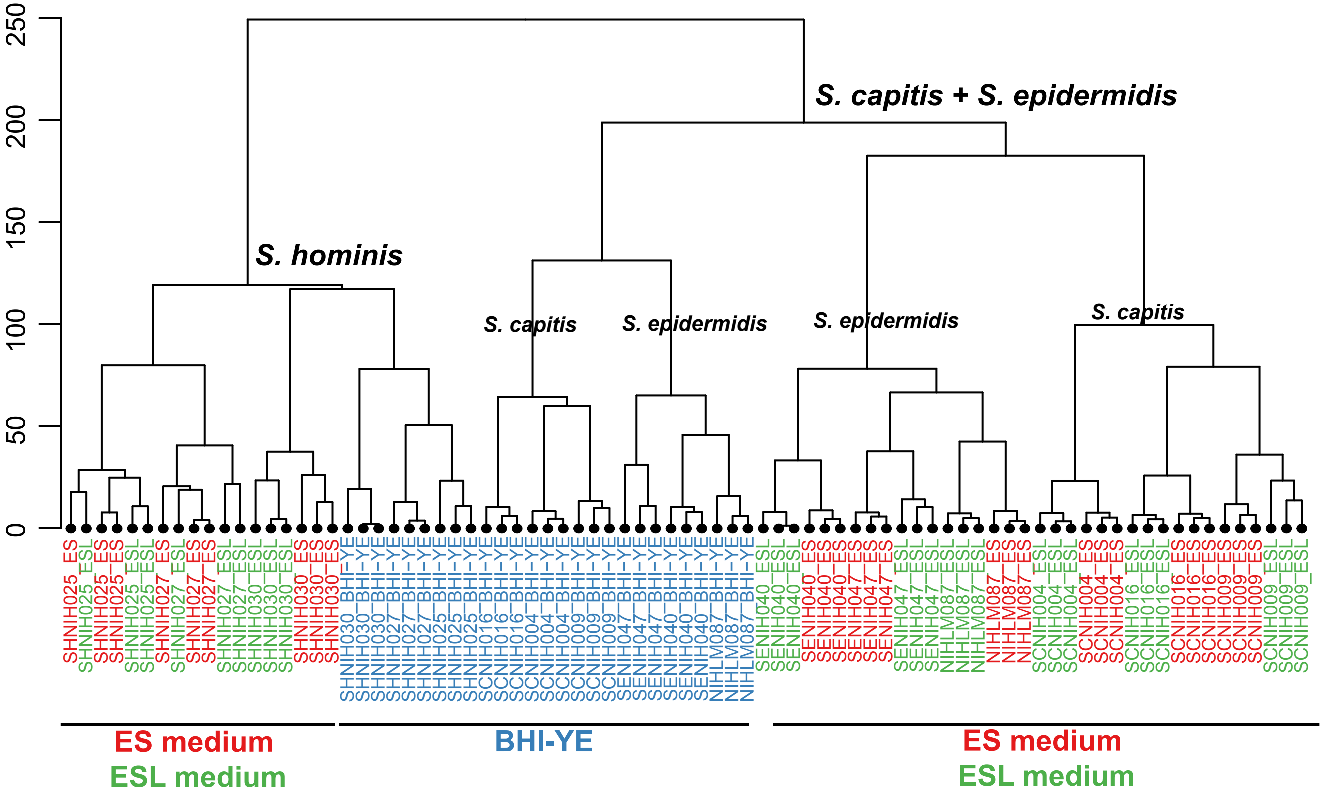
0
50
100
150
200
250
S. capitis + S. epidermidis
S. hominis
S. capitis
S. epidermidis
S. epidermidis
S. capitis
SHNIH025_ES
SHNIH025_ESL
SHNIH025_ES
SHNIH025_ES
SHNIH025_ESL
SHNIH025_ESL
SHNIH027_ES
SHNIH027_ESL
SHNIH027_ES
SHNIH027_ES
SHNIH027_ESL
SHNIH027_ESL
SHNIH030_ESL
SHNIH030_ESL
SHNIH030_ESL
SHNIH030_ES
SHNIH030_ES
SHNIH030_ES
SHNIH030_BHI-YE
SHNIH030_BHI-YE
SHNIH030_BHI-YE
SHNIH027_BHI-YE
SHNIH027_BHI-YE
SHNIH027_BHI-YE
SHNIH025_BHI-YE
SHNIH025_BHI-YE
SHNIH025_BHI-YE
SCNIH016_BHI-YE
SCNIH016_BHI-YE
SCNIH016_BHI-YE
SCNIH004_BHI-YE
SCNIH004_BHI-YE
SCNIH004_BHI-YE
SCNIH009_BHI-YE
SCNIH009_BHI-YE
SCNIH009_BHI-YE
SENIH047_BHI-YE
SENIH047_BHI-YE
SENIH047_BHI-YE
SENIH040_BHI-YE
SENIH040_BHI-YE
SENIH040_BHI-YE
NIHLM087_BHI-YE
NIHLM087_BHI-YE
NIHLM087_BHI-YE
SENIH040_ESL
SENIH040_ESL
SENIH040_ESL
SENIH040_ES
SENIH040_ES
SENIH047_ES
SENIH047_ES
SENIH047_ES
SENIH047_ESL
SENIH047_ESL
SENIH047_ESL
NIHLM087_ESL
NIHLM087_ESL
NIHLM087_ESL
NIHLM087_ES
NIHLM087_ES
NIHLM087_ES
SCNIH004_ESL
SCNIH004_ESL
SCNIH004_ESL
SCNIH004_ES
SCNIH004_ES
SCNIH004_ES
SCNIH016_ESL
SCNIH016_ESL
SCNIH016_ESL
SCNIH016_ES
SCNIH016_ES
SCNIH016_ES
SCNIH009_ES
SCNIH009_ES
SCNIH009_ES
SCNIH009_ESL
SCNIH009_ESL
SCNIH009_ESL
ES medium
ESL medium
BHI-YE
ES medium
ESL medium

Figure S9 Hierarchal clustering of variance stabilizing transformation-normalized reads for 1647 genus-core genes from all RNA-seq samples (Euclidean distance; Ward).

The x-axis represents sample clusters, and the y-axis represents the distances between samples. Colors depict different growth media. Red: ES, Green: ESL, and Blue is BHI-YE. Samples clustered by species (shown as labels) and by growth medium.

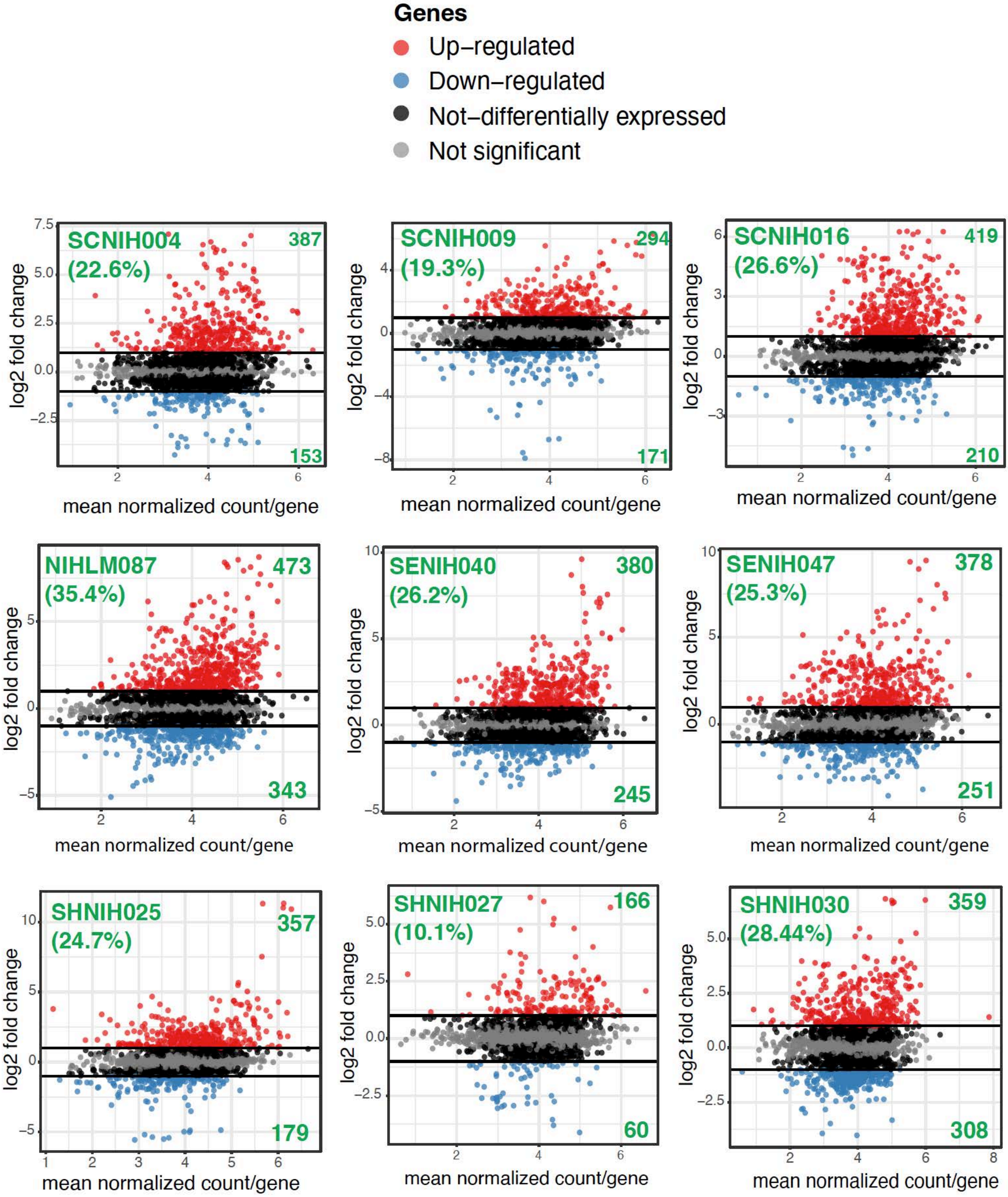
Genes
Up–regulated
Down–regulated
Not–differentially expressed
Not significant
SCNIH004 (22.6%) 387 153
SCNIH009 (19.3%) 294 171
SCNIH016 (26.6%) 419 210
NIHLM087 (35.4%) 473 343
SENIH040 (26.2%) 380 245
SENIH047 (25.3%) 378 251
SHNIH025 (24.7%) 357 179
SHNIH027 (10.1%) 166 60
SHNIH030 (28.44%) 359 308
log2 fold change
mean normalized count/gene

Figure S10 Differential gene expression plot for each isolate.

Each box represents the fold change in expression of each gene in the ES medium relative to BHI-YE for each isolate plotted against the mean normalized count per gene. Red dots represent upregulated genes, blue represent downregulated genes ($\geq$ or $\leq$ 2-fold change, adjusted P value < 0.05, DESeq). The actual number of upregulated (top) and downregulated (bottom) genes is show in green. The numbers shown in green inside brackets placed below each isolate label represent the percentage of genes within a genome that were differentially regulated in the ES medium relative to BHI-YE.

Supplementary Table 1 – Prevalence and mean relative abundance of skin-resident staphylococcal species based on 16S rRNA amplicon analysis.

Species	Mean Percent Relative Abundance* ± Standard Deviation	Prevalence by subjects (n = 22) (%)	Prevalence by samples (n = 298) (%)
S. epidermidis	52.25 ± 1.87	22 (100)	284 (95.30)
S. capitis	26.37 ± 1.77	22 (100)	225 (75.50)
S. hominis	23.65 ± 1.82	22 (100)	208 (69.80)
S. warneri	8.27 ± 1.23	22 (100)	121 (40.60)
S. lugdunensis	3.98 ± 1.69	17 (77)	31 (10.40)
S. haemolyticus	6.27 ± 1.49	16 (73)	53 (17.79)
S. auricularis	30.79 ± 7.03	13 (59)	27 (9.06)
S. cohnii	12.19 ± 3.25	13 (59)	35 (11.74)
S. pettenkoferi	9.19 ± 1.92	9 (41)	19 (6.38)
S. aureus	8.44 ± 2.83	8 (36)	17 (5.70)
S. saccahrolyticus	16.32 ± 3.47	6 (27)	31 (10.40)
S. epidermidis group	16.03 ± 2.88	6 (27)	27 (9.06)
S. saprophyticus	0.84 ± 0.28	6 (27)	8 (2.68)
S. simulans	3.31 ± 1.14	5 (23)	6 (2.01)
S. pasteuri	3.26 ± 1.06	5 (23)	14 (4.70)
S. haemolyticus group	4.48 ± 1.35	4 (18)	13 (4.36)
S. petrasi	0.86 ± 0.22	4 (18)	7 (2.35)

*Mean percent relative abundance of each species was calculated using only those samples that were positive for the given species (range: minimum 3 reads to maximum 12119 reads).

Supplementary Table 5 – Genus-core genes with predicted role in skin colonization

Gene ID	Genes	Function	Role in skin colonization
S0510	*srtA*	covalent anchoring of adhesins to the bacterial cell wall	bacterial adhesion, biofilm formation, and immune escape (1)
S1463, S0384, S0385, S1644	*dltABCD*	D-alanylation of teichoic acids	Resistance to host antimicrobial peptides (2)
S0319	*mprF*	phospholipid lysylation	Resistance to host antimicrobial peptides (3)
S0957	*sepA*	protease	AMP degradation (4)
S1750, S1576	*vraF, vraG*	AMP export	Resistance to host antimicrobial peptides (5)
S1493, S1721, S1590	*graR, graS, graX*	Aps system	AMP sensor, regulator of AMP resistance mechanisms (6)
S0518- S0520	*capABC*	Poly-γ-DL-glutamate capsule biosynthesis	Protects from AMPs, phagocytosis, and high salt concentration (7)
S1387	*oatA*	O-acetylates peptidoglycan	Lysozyme resistance (8)
S0663	*vraX*	binds host complement protein	Inhibit classical complement pathway (9)
S1611	*atlE*	Bifunctional autolysin/adhesin	biofilm formation, vitronectin binding (10)
S1620, S1526, S1030, S1031	*pmtABCD*	ABC transporter for all Phenol-soluble modulins (PSM) classes	Virulence, biofilm (11)
S0455, S1812	-	Phenol-soluble modulins-beta	promote biofilm maturation and dissemination (12)

S0624, S0625, S1613, S1766, S0626, S0627, S1813	*tagDXBGHA*	poly-glycerol-phosphate teichoic acids synthesis	Role in nasal colonization (13)
S1337	*gehC*	lipase	establish residence in the hair follicles (14)
S0557	*betA*	Choline dehydrogenase	biosynthesis of the osmoprotectant glycine betaine (15)
S0487, S0486, S0485, S1232	*opuCABCD*	Glycine betaine/choline/carnitine transport	Osmoprotolerance (16)
S1313, S0867-S0872<br>S0873	*ureABCEFGD*<br><br>*yut*	urease-production<br><br>urea transport	acid response and pH homeostasis (17)
S0947- S0949	*yfmCDE*	ferric citrate transporter	Iron acquisition (18)
S0951	*isdG*	liberates iron from host heme	Iron acquisition (19)

Supplementary Table 8. Composition of artificial skin media (www.pickeringlabs.com/)

1. **Eccrine Sweat (ES):**

Artificial Eccrine Perspiration (pH 5.5); Catalog Number: 1700-0023

Amino Acids
Concentrations for listed amino acids range from 0.002 g/L (for Taurine) to 0.30 g/L (for Serine)

- Glycine
- L-Alanine
- L-Arginine
- L-Asparagine
- L-Aspartic acid
- L-Citrulline
- L-Glutamic acid
- L-Histidine
- L-Isoleucine
- L-Leucine
- L-Lysine as hydrochloride
- L-Methionine
- L-Ornithine as hydrochloride
- L-Phenylalanine
- L-Serine (Largest amount)
- L-Threonine
- L-Tyrosine
- L-Valine
- Taurine

Metabolites
Concentration for listed metabolites range from 0.015 g/L (for Uric Acid) to 1.74 g/L (for Urea)

- Uric acid
- Urea
- Lactic acid
- Ammonia

Minerals

- Sodium – 33 mmole/L
- Zinc – 11.21 µmole/L
- Chloride – 80.34 mmole/L
- Calcium – 5.49 mmole/L
- Iron – 4.62 µmole/L
- Magnesium – 1.67 mmole/L
- Potassium – 33 mmole/L
- Sulfate – 2.57 mmole/L

2. **Eccrine Sweat with lipids (ESL):** This medium was prepared using ES medium as the base. Tween 80 was added at 0.1%. The following sebum/apocrine emulsion was added at 1% for final growth curves.

   Apocrine sweat: emulsion of sebum plus other ingredients. Catalog Number 1700-0556

**Compound**	**Concentration (g/L)**
L-Alanine	0.1-0.5
L-Aspartic acid	0.01-0.1
L-Citrulline	0.1-0.5
L-Glutamic acid	0.5 -2
L-Glutamine	0.1-0.5
Glycine	0.1-0.5
L-isoleucine	0.1-0.5
L-Leucine	0.1-0.5
L-Lysine	0.5-2
L-Phenylalanine	0.01-0.1
L-Proline	0.1-0.5
L-Serine	0.1-0.5
L-Threonine	0.1-0.5
L-Tryptophan	0.1-0.5
L-Tyrosine	0.01-0.1
L-Valine	0.1-0.5
Creatine	0.01-0.1
Urea	0.2-2
Citric acid	0.1-0.5
Formic Acid	0.01-0.1
Lactic Acid	0.5 - 2
Glucose	0.01-0.1
Butyric acid	2-3
Valeric acid	2-3
a-hydroxy-n-butyric acid-sodium salt	0.01-0.1
3-hydroxybutyric acid	0.01-0.1
a-hydroxy-iso-butyric acid	0.1-0.5
$(NH_4)_2SO_4$	0.1-0.5
$CaCl_2*2H_2O$	0.5 - 2
$CuSO_4*5H_2O$	0.001-0.01
$Fe(NO_3)_3*9H_2O$	0.001-0.01
$MgCl_2*6H_2O$	0.1-0.5
NaCl	0.5 - 2
$ZnCl_2$	0.001-0.01

Sebum components

- Palmitic acid (CAS# 57-10-3): 0.5 % w/v

- Stearic acid (CAS# 57-11-4): 0.25% w/v
- Oleic Acid (CAS# 112-80-1): 0.9 % w/v
- Linoleic acid (CAS# 60-33-3): 0.25% w/v
- Coconut oil (CAS# 8001-31-8): 0.75 % w/v
- Olive oil (CAS# 800-25-0): 1% w/v
- Paraffin Wax (CAS# 8002-74-2): 0.5 % w/v
- Synthetic Spermacetti: 0.75% w/v
- Squalene (CAS# 111-02-4): 0.25% w/v
- Cholesterol (CAS# 57-88-5): 0.25% w/v
- Triethanolamine (CAS#102-71-6): 0.8% w/v

Supplementary Table 9. Distribution of differentially expressed genes in ES medium relative to BHI-YE between core and accessory gene partitions

Differentially expressed genes = Fold change ≥ or ≤ 2, adjusted P value < 0.05

Species	Isolate	No. of genus-core genes	Percent (%)	No. of species-restricted core genes	Percent (%)	No. of accessory genes	Percent (%)
S. epidermidis	NIHLM087	583	72	149	18	81	10
	SENIH040	428	70	107	17	78	13
	SENIH047	398	64	119	19	104	17
S. capitis	SCNIH004	345	66	149	29	28	5
	SCNIH009	268	58	131	29	62	13
	SCNIH016	438	70	172	27	18	3
S. hominis	SHNIH025	371	70	70	13	92	17
	SHNIH027	166	74	32	14	26	12
	SHNIH030	463	70	85	13	115	17

Datasets in Excel format

Supplementary Table 2. Genome statistics of isolates used in the current study

Supplementary Table 3. Gene presence absence matrix based on individual species pan-genome

Supplementary Table 4. Gene presence absence matrix of merged genus pan-genome

Supplementary Table 6. List of species-restricted core genes detected in genus pan-genome.

Supplementary Table 7. pan-GWAS analysis showing clade-specific gene enrichment in each species

Supplementary Table 10. List of differentially expressed genes in the ES medium relative to BHI-YE per isolate

SI References

1. S. Cascioferro, M. Totsika, D. Schillaci, Sortase A: an ideal target for anti-virulence drug development. *Microb Pathog* **77**, 105-112 (2014).
2. A. Peschel *et al.*, Inactivation of the dlt operon in Staphylococcus aureus confers sensitivity to defensins, protegrins, and other antimicrobial peptides. *J Biol Chem* **274**, 8405-8410 (1999).
3. A. Peschel *et al.*, Staphylococcus aureus resistance to human defensins and evasion of neutrophil killing via the novel virulence factor MprF is based on modification of membrane lipids with l-lysine. *J Exp Med* **193**, 1067-1076 (2001).
4. Y. Lai *et al.*, The human anionic antimicrobial peptide dermcidin induces proteolytic defence mechanisms in staphylococci. *Mol Microbiol* **63**, 497-506 (2007).
5. M. Falord, G. Karimova, A. Hiron, T. Msadek, GraXSR proteins interact with the VraFG ABC transporter to form a five-component system required for cationic antimicrobial peptide sensing and resistance in Staphylococcus aureus. *Antimicrob Agents Chemother* **56**, 1047-1058 (2012).
6. M. Li *et al.*, The antimicrobial peptide-sensing system aps of Staphylococcus aureus. *Mol Microbiol* **66**, 1136-1147 (2007).
7. S. Kocianova *et al.*, Key role of poly-gamma-DL-glutamic acid in immune evasion and virulence of Staphylococcus epidermidis. *J Clin Invest* **115**, 688-694 (2005).
8. A. Bera, S. Herbert, A. Jakob, W. Vollmer, F. Gotz, Why are pathogenic staphylococci so lysozyme resistant? The peptidoglycan O-acetyltransferase OatA is the major determinant for lysozyme resistance of Staphylococcus aureus. *Mol Microbiol* **55**, 778-787 (2005).
9. J. Yan *et al.*, Staphylococcus aureus VraX specifically inhibits the classical pathway of complement by binding to C1q. *Mol Immunol* **88**, 38-44 (2017).
10. C. Heilmann, M. Hussain, G. Peters, F. Gotz, Evidence for autolysin-mediated primary attachment of Staphylococcus epidermidis to a polystyrene surface. *Mol Microbiol* **24**, 1013-1024 (1997).
11. S. S. Chatterjee *et al.*, Essential Staphylococcus aureus toxin export system. *Nat Med* **19**, 364-367 (2013).
12. R. Wang *et al.*, Staphylococcus epidermidis surfactant peptides promote biofilm maturation and dissemination of biofilm-associated infection in mice. *J Clin Invest* **121**, 238-248 (2011).
13. V. Winstel, P. Sanchez-Carballo, O. Holst, G. Xia, A. Peschel, Biosynthesis of the unique wall teichoic acid of Staphylococcus aureus lineage ST395. *mBio* **5**, e00869 (2014).
14. K. Nakamura, M. R. Williams, J. M. Kwiecinski, A. R. Horswill, R. L. Gallo, Staphylococcus aureus Enters Hair Follicles Using Triacylglycerol Lipases Preserved through the Genus Staphylococcus. *J Invest Dermatol* **141**, 2094-2097 (2021).
15. J. E. Graham, B. J. Wilkinson, Staphylococcus aureus osmoregulation: roles for choline, glycine betaine, proline, and taurine. *J Bacteriol* **174**, 2711-2716 (1992).

16. D. Casey, R. D. Sleator, A genomic analysis of osmotolerance in Staphylococcus aureus. *Gene* **767**, 145268 (2021).
17. C. Zhou *et al.*, Urease is an essential component of the acid response network of Staphylococcus aureus and is required for a persistent murine kidney infection. *PLoS Pathog* **15**, e1007538 (2019).
18. V. Braun, C. Herrmann, Docking of the periplasmic FecB binding protein to the FecCD transmembrane proteins in the ferric citrate transport system of Escherichia coli. *J Bacteriol* **189**, 6913-6918 (2007).
19. K. P. Haley, E. M. Janson, S. Heilbronner, T. J. Foster, E. P. Skaar, Staphylococcus lugdunensis IsdG liberates iron from host heme. *J Bacteriol* **193**, 4749-4757 (2011).
